# EPIsHilbert: Prediction of Enhancer-Promoter Interactions via Hilbert Curve Encoding and Transfer Learning

**DOI:** 10.3390/genes12091385

**Published:** 2021-09-06

**Authors:** Mingyang Zhang, Yujia Hu, Min Zhu

**Affiliations:** Department of Computer Science, Sichuan University, Chengdu 610065, China; 15208471128@163.com (M.Z.); dorisjia0121@163.com (Y.H.)

**Keywords:** Hilbert curve, enhancer-promoter interactions, transfer learning

## Abstract

Enhancer-promoter interactions (EPIs) play a significant role in the regulation of gene transcription. However, enhancers may not necessarily interact with the closest promoters, but with distant promoters via chromatin looping. Considering the spatial position relationship between enhancers and their target promoters is important for predicting EPIs. Most existing methods only consider sequence information regardless of spatial information. On the other hand, recent computational methods lack generalization capability across different cell line datasets. In this paper, we propose EPIsHilbert, which uses Hilbert curve encoding and two transfer learning approaches. Hilbert curve encoding can preserve the spatial position information between enhancers and promoters. Additionally, we use visualization techniques to explore important sequence fragments that have a high impact on EPIs and the spatial relationships between them. Transfer learning can improve prediction performance across cell lines. In order to further prove the effectiveness of transfer learning, we analyze the sequence coincidence of different cell lines. Experimental results demonstrate that EPIsHilbert is a state-of-the-art model that is superior to most of the existing methods both in specific cell lines and cross cell lines.

## 1. Introduction

Promoters and enhancers are two important cis-regulatory elements that control gene transcription [1]. Enhancers [2,3] can increase the transcription of specific genes and promoters [4,5], and determine the position of transcription start point and frequency. Enhancer–promoter interactions (EPIs) are vital for the regulation of gene expression [6] and reveal associations between some special genes and diseases [7,8,9]. For example, Smeno et al. [10] found that there are EPIs existing in introns of FTO and Irx3 with increased risk for obesity and type-2 diabetes.

In recent decades, many studies [11,12,13,14,15,16] have shown that the mechanism of EPIs is complicated: one enhancer can act on one or more target promoters, while one target promoter can be co-regulated by one or more enhancers. Although the experimental approaches can identify EPIs accurately, such as FISH [17] and chromosome conformation capture (3C) [18], the result may contain a lot of irrelevant information. Moreover, experimental approaches are also expensive and time consuming.

Therefore, with the development of various high-throughput technologies, computational methods have become usual alternatives for identifying EPIs. Whalen et al. [19] proposed a model named TargetFinder, which used lots of sequence and genomic information obtained in biological experiments to predict EPIs. Talukder et al. [13] built an Adaboost model using functional and genomic data to predict EPIs. However, these traditional machine learning methods often require feature engineering, which leads to redundant features, such as GTB [14], GBRT [15], and random forest [16]. The fact that potential information may affect EPI predictions is excluded from consideration.

In recent years, deep learning methods have been proposed to address the above limitations, which build different neural network architectures to learn potential sequence information. One-hot and its variant high-order encoding are usual encoding methods [20,21,22,23] that pay more attention to extract sequence context information than the potential spatial position information contained in a sequence. For example, Zhuang et al. [21] used one-hot to encode enhancer and promoter sequences that only consider the information of single nucleotides. In order to learn more relevant sequence information, many methods use CNN (Convolutional Neural Networks) to extract potential sequence information, such as EPIsCNN [21] and EPIVAN [6]. Singh et al. [20] proposed SPEID, a hybrid of CNN and LSTM (Long short-term Memory) that better characterizes long-range interactions. However, LSTM may cause a long operation time. Min et al. [22] used a matching heuristic from natural language inference to explore the interaction between enhancers and promoters. Although these methods found different ways to obtain more information on EPIs, they still ignored the spatial position relationship.

Overall, even though these researchers have made considerable progress, some limitations still exist. First, almost all these methods do not take into consideration the spatial position relationship. Therefore, these models cannot extract more information. Second, few studies have focused on the analysis of features extracted by model from the spatial perspective; they only use the quantitative value of AUC (area under the curve) and AUPR (area under the precision–recall curve) to evaluate prediction accuracy. Third, most recent methods lack generalization capability across different cell line datasets. They obtained satisfactory prediction accuracy when train datasets were the same as test datasets, but performed worse across cell lines. Although EPIHC [23] and SEPT [24] both use different transfer learning approaches, their results are unsatisfactory.

To address the above limitations, we have designed a model named EPIsHilbert using Hilbert curve encoding and transfer learning. Many researchers have shown that enhancers interact with promoters through complex spatial positions, such as rotating or folding around. Hilbert curve encoding avoids some loss of the spatial position relationship between an enhancer and a promoter, so it can help improve a model’s performance. In order to explore the sequence features that affect the EPIs and their spatial relationships, we add a class activation map (CAM) visualization to display the frequency of sequence features. The occurrence of some diseases is usually related to genetic elements that control the gene regulation, so sequence features with a high frequency of occurrence can be further used in genetic testing or disease diagnosis. For achieving satisfactory prediction performance across cell lines, we proposed two transfer learning strategies to pre-train the model with data from various cell lines. We further explored the reasons for the conclusions of transfer learning according to the analysis of coincidence degree. Experiment results demonstrated that EPIsHilbert not only improved the accuracy of the model prediction in some target cell lines, but also obtained satisfying performance across cell lines.

## 2. Methods and Materials

### 2.1. Datasets

In this study, we used the same dataset from TargetFinder [19] and SPEID [20] as the original EPIs dataset, consisting of six cell lines: GM12878, HUVEC, HeLa-S3, K562, NHEK, and IMR90. The dataset of each cell line contains enhancer sequences, promoter sequences, positive samples, and negative samples of EPIs. Enhancer and promoter sequences were derived from the Encyclopedia of DNA elements and filtered by the Roadmap Epigenomics Project [25]. Based on the Hi-C technology [26], EPIs could be detected.

All sequences were unified into a fixed length. The length of enhancer sequences is 3000 base pairs (bp), while that of the promoter is 2000 bp. In each cell line, the ratio of negative samples to positive samples is 20:1. Thus, for our dataset, a class imbalance problem existed. We used two methods, over-sampling and under-sampling, to achieve a balanced dataset. The details of the balanced dataset are shown in Table 1.

### 2.2. EPIsHilbert

Here, we proposed a prediction model called EPIsHilbert, using Hilbert curve to encode enhancer and promoter sequences, combining sequence analysis with deep learning and utilizing transfer learning strategies to pre-train the model. The overview of EPIsHilbert contains three main steps: Hilbert curve encoding, network architecture, and transfer learning pre-training.

#### 2.2.1. Hilbert Curve Encoding

**Figure 1:**
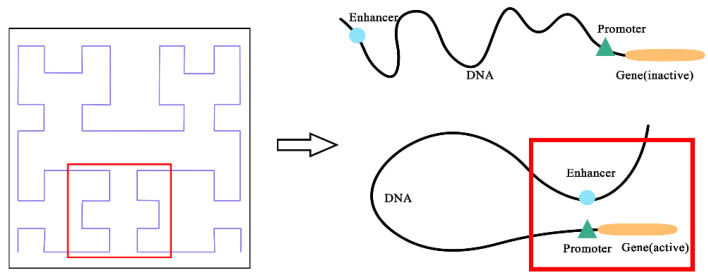
The relationship between Hilbert curve and Enhancer–promoter interactions (EPIs).

To address the limitation of ignoring spatial position relationships, we propose Hilbert curve encoding [27], a classic space-filling curve to encode enhancer and promoter sequences. As shown in Figure 1, the enhancer interacts with the promoter through complex spatial relationships, such as rotating or folding around. The Hilbert curve represents the interaction between enhancer and promoter by mapping the spatial interaction locations. It is clear that the Hilbert curve encoding reflects the actual interaction, though the enhancers and target promoters are close in space but distant from each other in position.

**Figure 2:**
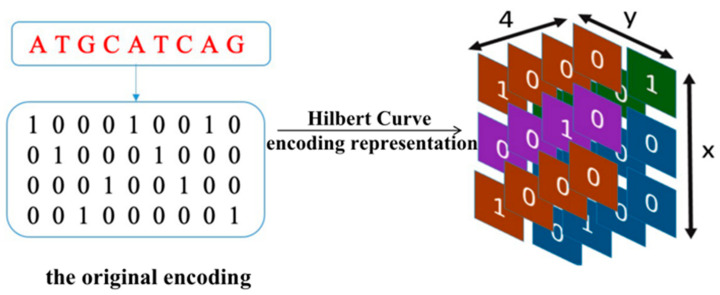
Hilbert curve encoding.

When using Hilbert curve encoding, as the vector space gradually transforms from low to high-dimensional space, the specified points on the line gradually tend towards a more exact point in the vector space. Therefore, Hilbert curve can convert a one-dimensional sequence into a three-dimensional matrix-vector, as we can see in Figure 2, representing enhancer–promoter long-range interaction and spatial position information. The process of Hilbert curve encoding is as follows:

i. Unify the representation of DNA bases: ‘A’ is encoded as [1,0,0,0], ‘T’ is encoded as [0,1,0,0], ‘C’ is encoded as [0,0,1,0] and ‘G’ is encoded as [0,0,0,1].
ii. Confirm the Hilbert curve dimension.
iii. The Hilbert representation of sequence: for each base in the sequence, we encode the sequence in order of the Hilbert space to obtain a three-dimensional vector.

#### 2.2.2. Network Architecture

**Figure 3:**
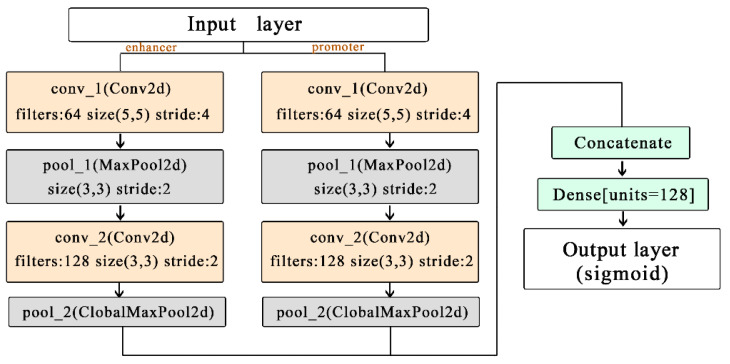
The relationship between Hilbert curve and EPIs.

After Hilbert curve encoding, the obtained three-dimensional vector can be regarded as an image with multiple channels. As such, we use a simple CNN network architecture as our prediction model to extract sequence features. As shown in Figure 3, the network architecture is composed of two convolutional layers, two pooling layers, and one connected layer. We split all samples of each cell line into a training set, a validation set, and a test set, with the ratio of 8:1:1. The parameters of the model were chosen with the learning rate 3 × 10^−4^ batch-size 100, and epochs 100. To prevent overfitting, we used early stopping to control the number of epochs and dropouts.

#### 2.2.3. Transfer Learning

Some studies show that using the data of one specific cell line provides a more accurate prediction of EPIs on this cell line than on the other five cell lines. This indicates that the ability to predict EPIs across cell lines is not good. Rao et al. [28] reported that there are 55–75% DNA interactions sharing different cell lines, so there are common features among them. Assuming that a common motif in various cell lines can facilitate E–P interaction, transfer learning means that we incorporate more features as training sets. To further improve the prediction performance of the model across cell lines and explore the correlation among the six cell lines, we propose two transfer learning methods. Transfer learning [29] is the improvement of learning in a new task through the transfer of knowledge from a related task that has already been learned. Additionally, using transfer learning will increase the sample size, perhaps leading to more stable parameter estimates.

**Using the Data from Other Cell Lines to Pre-Train a Model.** We propose a method of transfer learning by using data from other cell lines. For predicting a specific cell line, after using data from all other cell lines to pre-train the CNN model, we use the cell line-specific data to fully train the model. For any given specific cell line, the transfer learning process is as follows:

i. Take the specific cell line as a new cell line, $D^{new}$.
ii. Pre-train the CNN model with the training set, denoted as $D^{all-new}$, from other five cell lines for 6–8 epochs.
iii. Train the model on training set $D^{new}$ for 10–15 epochs.
iv. Evaluate the model on $D_{test}^{new}$.

**Using the Data from All Cell Lines to Pre-Train a Model.** The second transfer learning strategy we propose is in a strong form, which uses the data from all cell lines to pre-train the model. After pre-training, the convolution layer and the pooling layer are frozen. The subsequent training process no longer learns new features, and only adjusts the model parameters for the target cell line. Note that the training, validation and test sets were generated in the way described in Section 2.2.2. This transfer learning process is as follows:

i. Pre-train the CNN model with the training set from all six cell lines or 6–8 epochs.
ii. Freeze the convolution layer and pooling layer of enhancer and promoter branches.
iii. Train the model on the training set from the specific cell line for 10–15 epochs.
iv. Evaluate the model on the test set.

### 2.3. Evaluation Metrics

EPI prediction is equivalent to a binary classification of sequence prediction where three classification indicators are commonly used: precision, recall, and F1-score. Due to the imbalanced datasets, the performance of our model is assessed by area under the precision–recall curve (AUPR) and receiver operating characteristic curve (AUC). AUPR is the area under the precision–recall curve, reflecting trends between precision and recall, better if close to 1. Similarly, AUC is plotted with false positive rate as the horizontal axis and sensitivity as the vertical axis. Thus, AUC reaches its best value at 1 and its worst at 0.

## 3. Results

### 3.1. The Prediction Performance of Cell Line-Specific Model

In this section, we demonstrate that the model trained on the specific cell line alone is not applicable to other cell lines. The results of the model on each cell line in terms of AUC and AUPR are shown in Table 2 and Table 3, respectively. On the whole, the cell line-specific model that uses the same cell line in training and test sets performs very well. Practically speaking, the AUC and AUPR values are both over 0.9, even up to 0.983 and 0.988, separately. As seen from the non-diagonal results of tables, the cross-cell line ability of the model is significantly bad. For example, when we use the model fitted for cell line NHEK to predict EPIs for the other five cell lines, the AUPR values of the other five cell lines range from 0.465 to 0.550, while that of NHEK is 0.988.

We can draw two conclusions based on the above results. One, the model without transfer learning lacks a generalization capability across different cell line datasets. On the other hand, the model trained on one specific cell line has a good predictive ability in that cell line.

### 3.2. Advantages of Transfer Learning

#### 3.2.1. Using the Data from Other Cell Lines to Pre-Train a Model

The model transferred to a new cell line in this section is called EPIsHilbert-transOne. Table 4 shows the AUPR value of EPIsHilbert-transOne on each cell line. At the same time, we add EPIsHilbert for comparison. The performance of the cross-cell line prediction is significantly improved. For instance, when we used the cell line-specific model fitted for cell line NHEK to predict EPIs for the other five cell lines, the method yielded AUC values of 0.441–0.557, much smaller than 0.887–0.931 obtained from EPIsHilbert-transOne. After using transfer learning, the F1 value increased by at least 0.66, and the AUC and AUPR values also increased by more than 0.40. For cell line-specific predictions, there is a significant improvement for HUVEC, K562, GM12878, and Hela-S3, while EPIsHilbert-transOne performs slightly worse than EPIsHilbert on NHEK and IMR90.

Hence, our proposed EPIsHilbert-transOne clearly outperforms EPIsHilbert across cell lines and also improves the performance on four cell lines for cell line-specific predictions.

#### 3.2.2. Using the Data from All Cell Lines to Pre-Train a Model

**Figure 4:**
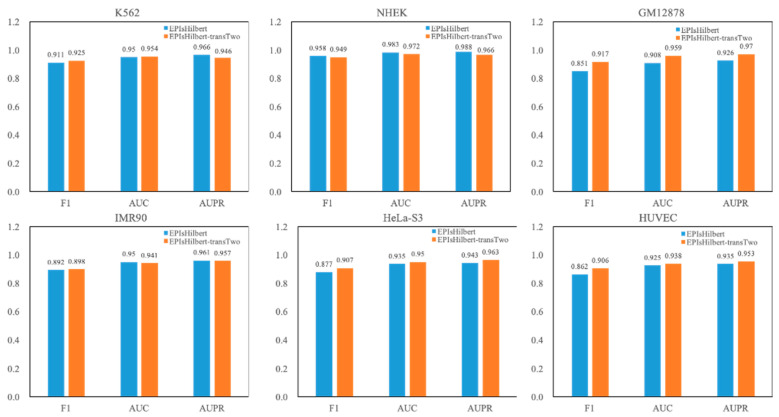
The comparison between EPIsHilbert and EPIsHilbert-transTwo.

For the convenience of discussion, our model in this section is denoted as EPIsHilbert-transTwo. The experimental result of EPIsHilbert-transTwo on each cell line in terms of AUPR is shown in Table 5. It is observed that EPIsHilbert-transTwo improves the performance of the model across cell lines. After using transfer learning, the F1 value is increased at least 0.69, the AUC and AUPR values are also increased more than 0.46. As seen in Figure 4, comparing EPIsHilbert-transTwo with EPIsHilbert, we can find that, for cell line-specific predictions, there is a significant improvement in HUVEC, GM12878, and Hela-S3, while EPIsHilbert-transTwo performs slightly worse than EPIsHilbert on NHEK, IMR90, and K562.

#### 3.2.3. The Analysis of Epis Overlap Ratio for Different Cell Lines

After using the strategy of transfer learning, the prediction ability of the model across cell lines was improved, which indicated there may have been common sequence fragments among all cell lines. To demonstrate this hypothesis, we explore the sequence overlap ratio of EPIs between different cell lines. The overlap ratio refers to the sequence similarity of EPIs in different cell lines, and the calculation process is as follows:

i. Set the number of coincident samples $num_{i}$(i∈ [1,5]) and select each enhancer–promoter pair $E_{t}\cdot P_{t\;}\left(t\in \left[1,\;m\right]\right)$. The m represents EPIs positive samples size in the target cell line.
ii. Take the other five cell lines as a dataset, $dataset_{i}$. Search each enhancer–promoter pair $E_{ij}\cdot P_{ij\;}\left(j\in \left[1,\;n\right]\right)$ in EPIs positive samples of $dataset_{i}$. The n represents EPIs positive samples size in $dataset_{i}$.
iii. For each $E_{t}\cdot P_{t\;}$, search all $E_{ij}\cdot P_{ij\;}$ and calculate their overlap ratio. If the overlap ratio $\geq \omega_{0}$, then the number of n plus one. $\omega_{0}$ is the threshold of overlap ratio.
iv. Calculate the remaining four cell lines in a loop to obtain the number of overlapping EPIs between the target cell line and the other five cell lines.
v. Modify the target cell line and repeat the above steps five times to complete the overlap number statistics.

$\omega_{0}$ is set to 100% and 80%, respectively. The results are shown in Table 6 and Table 7. When $\omega_{0}$ is 100%, it can be seen that the overlapping number of EPIs from NHEK and IMR90 completely with each cell line is small, while the other four cell lines have more overlapping EPIs. When $\omega_{0}$ is reduced to 80%, the number of EPIs from NHEK and IMR90 does not increase much. However, the other four cell lines show a hefty increase, with the largest increase of more than 40 pairs of similar EPIs.

Therefore, we can infer that, when $\omega_{0}$ continues to decrease, the four cell lines, except NHEK and IMR90, will increase even more. NHEK and IMR90 have more particular features. On the whole, EPIs in different cell lines have some common sequence features. Thus, we prove the effectiveness of transfer learning.

### 3.3. Model Comparison

To consolidate the importance of our study, we compared the performance of our proposed models with the other two typical baseline predictors. The two typical baseline predictors are SPEID [20] and EPIsCNN [6]. SPEID, a hybrid of CNN and LSTM, only uses sequence data for prediction, while EPIsCNN uses a simple CNN structure that is the same as SPEID. As shown in Table 8, the experimental result shows that the three methods we proposed models all outperform SPEID and EPIsCNN.

These three models we proposed are suitable for different cell lines. To be specific, EPIsHilbert achieves slightly better performance on NHEK and IMR90, while EPIsHilbert-transOne performs better on K562 and Hela-S3. Moreover, on HUVEC and GM12878 cell lines, we obtain the best prediction result from the EPIsHilbert-transTwo model.

Based on the above results, we can infer that NHEK and IMR90 cell lines have more unique features because the results are not ideal. However, the two models using transfer learning perform better on K562, HeLa-S3, HUVEC, and GM12878 cell lines. It suggested that many features that influence EPIs in these four cell lines are also shared in all six cell lines.

### 3.4. Visulation of Sequences Features and Their Relationship

In our work, the proposed class activation map (CAM) [30] visualized the three-dimensional vectors with a heat map that presents the spatial distribution of features, and color depth indicates the influence of features on the interactions of enhancers and promoters.

**Figure 5:**
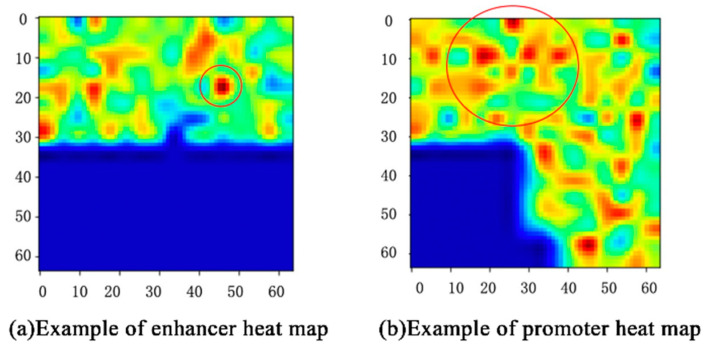
Example of CAM.

For example, the CAM of one enhancer–promoter pair in IMR90 is shown in Figure 5. The features that play an important role in EPIs are mainly concentrated in the upper half of Figure 5. The deeper the red, the greater the impact. Likewise, features in the lower-left corner almost do not influence EPIs. For each input enhancer–promoter pair, CAM can not only help us observe the distribution of the features of the EPIs from the space perspective, but also show the degree to which these features influence EPIs through the color-depth in the heat map. What is more, it also demonstrates that the enhancer approaches the promoter in complex spatial structures intuitively.

On the basis of CAM, we combine the heat map with the actual coding sequence to complete statistics on the frequency of features. We analyzed the heat map of enhancer and promoter sequences in 1254 EPIs positive samples of IMR90 cell lines to obtain the representations of features sequence. The statistics of enhancer and promoter feature occurrence frequency in IMR90 are shown in Table 9 and Table 10, respectively. GGG, AAA, CGT, CAT, and ATT are the features of enhancers with greater influence on EPIs. The features of promoters including CCC, TCC, TTC, TTT, and CTT have a greater impact on EPIs. It is obvious that there are high frequencies of only 3–4 nucleotide-long features, perhaps longer motifs may not have biological significance. As we all know, DNA transcription is based on three nucleotides. Thus, we can judge the motif that has a specific function as being 3–4 nucleotides long. However, our subsequent study will also extend the length to consider association-specific motifs and deeper analysis about diseases.

In this section, we counted the frequency of feature occurrences. The prevalence of some diseases is usually related to genetic elements that control gene regulation. Thus, in the field of disease diagnosis, features with a high frequency of occurrence can be used in genetic testing or to study the causes of diseases as an assisting technique.

## 4. Conclusions

In this article, we proposed a model named EPIsHilbert using only sequence data to predict EPIs. Being different from existing methods, EPIsHilbert is innovative at using the Hilbert curve to encode enhancer and promoter sequences and better preserves the spatial structure of the sequence. Thus, we can utilize sequences information and then obtain more details from them. Experimental results on six cell lines indicated that EPIsHilbert performs better than any existing method with an 0.908~0.983 of AUROC and 0.926~0.988 of AUPR.

In order to improve the ability of crossing cell lines, we used two transfer learning strategies to pre-train a CNN model by taking advantage of the data from various cell lines. Applied to the same CNN model, each method is more accurate for cross-cell line prediction than current practice, even if it may lose the specificity of each cell line. We further conducted a similarity analysis of the interactions between enhancers and promoters among cell lines. The result shows that NHEK and IMR90 cell lines contain more specific features, while the other four cell lines have more common features, so the prediction accuracy could be further improved by using transfer learning to pre-train the model.

We also created a class activation map to explore the features that affect EPIs and the spatial relationships of these features. On this basis, this paper combines a heat map with actual coding sequences to complete statistics regarding the frequency of features. The features with a high frequency of occurrence have a greater impact on the interactions between enhancers and promoters, and they can be further used in a genetic test for disease diagnosis and treatment.

Given the excellent performance of EPIsHilbert, we will continue to improve our approach. We may further explore other deep learning architecture for prediction, such as GCN. Then, we suggest that using EPIs data from more datasets may result in a better performance. Since we only use sequence data, integrating sequence data with epi-genomic data is an expected way to improve performance. Moreover, we expect EPIsHilbert can play an important role in all kinds of sequence prediction tasks.

## Figures and Tables

**Table 1:** The details of the balanced dataset.

Dataset	True EPIs	Aug True EPIs	False EPIs	Sub-Sample False EPIs
K562	1977	39,540	39,500	1977
GM12878	2113	42,260	42,200	2113
HeLa-S3	1740	34,800	34,800	1740
HUVEC	1524	30,480	30,400	1524
NHEK	1291	25,820	25,600	1291
IMR90	1254	25,080	25,000	1254

**Table 2:** AUC (area under the curve) value of cell line-specific model in different cell lines.

Test\Train Cell Line	K562	GM12878	HeLa-S3	HUVEC	NHEK	IMR90
K562	0.950	0.521	0.475	0.475	0.528	0.508
GM12878	0.497	0.908	0.526	0.609	0.472	0.524
HeLa-S3	0.479	0.508	0.935	0.467	0.557	0.508
HUVEC	0.541	0.492	0.573	0.925	0.441	0.481
NHEK	0.414	0.523	0.540	0.519	0.983	0.599
IMR90	0.496	0.460	0.471	0.484	0.526	0.950

**Table 3:** AUPR (area under the precision–recall curve) value of cell line-specific model in different cell lines.

Test\Train Cell Line	K562	GM12878	HeLa-S3	HUVEC	NHEK	IMR90
K562	0.966	0.511	0.494	0.471	0.515	0.495
GM12878	0.493	0.926	0.517	0.600	0.490	0.506
HeLa-S3	0.472	0.509	0.943	0.487	0.539	0.506
HUVEC	0.505	0.505	0.555	0.935	0.465	0.506
NHEK	0.481	0.520	0.544	0.514	0.988	0.611
IMR90	0.499	0.467	0.492	0.508	0.550	0.961

**Table 4:** AUPR value in EPIsHilbert-transOne.

Test\Train Cell Line	K562	GM12878	HeLa-S3	HUVEC	NHEK	IMR90
K562	0.981	0.870	0.868	0.860	0.889	0.893
GM12878	0.861	0.946	0.847	0.794	0.931	0.854
HeLa-S3	0.879	0.864	0.970	0.839	0.921	0.864
HUVEC	0.877	0.901	0.877	0.949	0.887	0.920
NHEK	0.908	0.861	0.913	0.856	0.982	0.753
IMR90	0.956	0.900	0.926	0.897	0.909	0.944

**Table 5:** AUPR value in EPIsHilbert-transTwo.

Test\Train Cell Line	K562	GM12878	HeLa-S3	HUVEC	NHEK	IMR90
K562	0.946	0.942	0.968	0.921	0.948	0.891
GM12878	0.942	0.970	0.938	0.927	0.931	0.910
HeLa-S3	0.922	0.936	0.963	0.920	0.957	0.939
HUVEC	0.942	0.929	0.944	0.953	0.953	0.894
NHEK	0.947	0.941	0.943	0.895	0.966	0.886
IMR90	0.959	0.941	0.929	0.934	0.863	0.957

**Table 6:** The analyses of EPIs overlap ratio for different cell lines (100%).

	Total EPIs	NHEK	IMR90	HUVEC	K562	GM12878	HeLa-S3
Total EPIs	—	1291	1254	1524	1977	2113	1740
NHEK	1291	—	42	51	33	19	63
IMR90	1254	42	—	41	11	25	15
HUVEC	1524	51	41	—	33	28	62
K562	1977	33	11	33	—	28	43
GM12878	2113	19	25	28	28	—	26
HeLa-S3	1740	63	15	62	43	26	—

**Table 7:** The analyses of EPIs overlap ratio for different cell lines (80%).

	Total EPIs	NHEK	IMR90	HUVEC	K562	GM12878	HeLa-S3
Total EPIs	—	1291	1254	1524	1977	2113	1740
NHEK	1291	—	42	63	52	22	85
IMR90	1254	42	—	48	15	32	18
HUVEC	1524	63	48	—	39	56	107
K562	1977	52	15	39	—	50	67
GM12878	2113	22	32	56	50	—	43
HeLa-S3	1740	85	18	107	67	43	—

**Table 8:** The comparison of AUPR value in different methods.

Test\Train Cell Line	K562	GM12878	HeLa-S3	HUVEC	NHEK	IMR90
SPEID [20]	0.809	0.782	0.829	0.778	0.845	0.764
EPIsCNN [6]	0.823	0.791	0.845	0.798	0.863	0.784
EPIsHilbert	0.966	0.926	0.943	0.935	0.988	0.961
EPIsHilbert-transOne	0.981	0.946	0.970	0.949	0.982	0.944
EPIsHilbert-transTwo	0.946	0.970	0.963	0.953	0.966	0.957

**Table 9:** The statistics of enhancer feature occurrence frequency in IMR90.

Feature	Frequency	Feature	Frequency	Feature	Frequency
GGG	924	TAT	637	GGGG	260
AAA	907	AAT	638	AAAA	227
CGT	740	GCG	625	CCGT	154
CAT	714	CCA	623	ATAT	152
ATT	677	AGC	606	AAAT	148
CGG	659	GTT	592	CCCA	139
ATA	657	TAA	589	TAAA	139

**Table 10:** The statistics of promoter feature occurrence frequency in IMR90.

Feature	Frequency	Feature	Frequency	Feature	Frequency
CCC	967	AAA	612	CCCC	293
TCC	734	CGC	594	TCTT	212
TTC	694	ATT	542	TCCC	199
TTT	688	CTC	529	CCGC	185
CTT	626	TAT	527	TTCT	175
TCT	624	CAT	515	CTTC	168
CCG	624	ATC	510	TTAT	158

## Data Availability

The source code are available here: https://github.com/zmyqx/E-HilbertEPIs, accessed on 1 March 2021.

## References

[1] Xu H., Zhang S., Yi X., Plewczynski D., Li M.J. (2020). Exploring 3D chromatin contacts in gene regulation: The evolution of approaches for the identi-fication of functional enhancer-promoter interaction. Comput. Struct. Biotechnol. J..

[2] Shlyueva D., Stampfel G., Stark A. (2014). Transcriptional enhancers: From properties to genome-wide predictions. Nat. Rev. Genet..

[3] Pennacchio L.A., Bickmore W., Dean A., Nobrega M.A., Bejerano G. (2013). Enhancers: Five essential questions. Nat. Rev. Genet..

[4] Lenhard B., Sandelin A., Carninci P. (2012). Metazoan promoters: Emerging characteristics and insights into transcriptional regulation. Nat. Rev. Genet..

[5] Sanyal A., Lajoie B.R., Jain G., Dekker J. (2012). The long-range interaction landscape of gene promoters. Nature.

[6] Hong Z., Zeng X., Wei L., Liu X. (2020). Identifying enhancer–promoter interactions with neural network based on pre-trained DNA vectors and attention mechanism. Bioinformatics.

[7] Jiang R. (2015). Walking on multiple disease-gene networks to prioritize candidate genes. J. Mol. Cell Biol..

[8] Schoenfelder S., Fraser P. (2019). Long-range enhancer–promoter contacts in gene expression control. Nat. Rev. Genet..

[9] Zeng W., Min X., Jiang R. (2019). EnDisease: A manually curated database for enhancer-disease associations. Database.

[10] Smemo S., Tena J.J., Kim K.H., Gamazon E.R., Sakabe N.J., Gómez-Marín C., Aneas I., Credidio F.L., Sobreira D.R., Wasserman N.F. (2014). Obesity-associated variants within FTO form long-range functional connections with IRX3. Nature.

[11] Ibrahim D.M., Mundlos S. (2020). The role of 3D chromatin domains in gene regulation: A multi-facetted view on genome organiza-tion. Curr. Opin. Genet. Dev..

[12] Zhang Y., Wong C.H., Birnbaum R.Y., Li G., Favaro R., Ngan C.Y., Lim J., Tai E., Poh H.M., Wong E. (2013). Chromatin connectivity maps reveal dynamic promoter–enhancer long-range as-sociations. Nature.

[13] Talukder A., Saadat S., Li X., Hu H. (2019). EPIP: A novel approach for condition-specific enhancer–promoter interaction prediction. Bioinformatics.

[14] Yang Y., Zhang R., Singh S., Ma J. (2017). Exploiting sequence-based features for predicting enhancer–promoter interactions. Bioinformatics.

[15] Zeng W., Wu M., Jiang R. (2018). Prediction of enhancer-promoter interactions via natural language processing. BMC Genom..

[16] Zhang T., Wang Y. An Approach for Recognition of Enhancer-promoter Associations based on Random Forest. Proceedings of the 2019 4th International Conference on Biomedical Signal and Image Processing (ICBIP 2019)-ICBIP 19.

[17] Trask B.J. (1991). Fluorescence in situ hybridization: Applications in cytogenetics and gene mapping. Trends Genet..

[18] Dekker J., Rippe K., Dekker M., Kleckner N. (2002). Capturing chromo G some conformation. Science.

[19] Whalen S., Truty R.M., Pollard K.S. (2016). Enhancer–promoter interactions are encoded by complex genomic signatures on looping chromatin. Nat. Genet..

[20] Singh S., Yang Y., Póczos B., Ma J. (2019). Predicting enhancer-promoter interaction from genomic sequence with deep neural networks. Quant. Biol..

[21] Zhuang Z., Shen X., Pan W. (2019). A simple convolutional neural network for prediction of enhancer–promoter interactions with DNA sequence data. Bioinformatics.

[22] Min X., Ye C., Liu X., Zeng X. (2020). Predicting enhancer-promoter interactions by deep learning and matching heuristic. Brief. Bioinform..

[23] Liu S., Xu X., Yang Z., Zhao X., Zhang W. (2020). EPIHC: Improving Enhancer-Promoter Interaction Prediction by using Hybrid features and Com-municative learning. arXiv.

[24] Jing F., Zhang S.W., Zhang S. (2020). Prediction of enhancer–promoter interactions using the cross-cell type information and domain adversarial neural network. BMC Bioinform..

[25] Bernstein B.E., Stamatoyannopoulos J.A., Costello J.F., Ren B., Milosavljevic A., Meissner A., Kellis M., Marra M.A., Beaudet A.L., Ecker J.R. (2010). The NIH roadmap epigenomics mapping consortium. Nat. Biotechnol..

[26] Belton J.M., McCord R.P., Gibcus J.H., Naumova N., Zhan Y., Dekker J. (2012). Hi–C: A comprehensive technique to capture the conformation of genomes. Methods.

[27] Anjum M.M., Tahmid I.A., Rahman M.S. (2019). CHilEnPred: CNN model with Hilbert curve representation of DNA sequence for enhancer prediction. BioRxiv.

[28] Rao S.S., Huntley M.H., Durand N.C., Stamenova E.K., Bochkov I.D., Robinson J.T., Sanborn A.L., Machol I., Omer A.D., Lander E.S. (2014). A 3D map of the human genome at kilobase resolution reveals principles of chromatin looping. Cell.

[29] Torrey L., Shavlik J. (2010). Transfer learning[M]//Handbook of research on machine learning applications and trends: Algorithms, methods, and techniques. IGI Glob..

[30] Selvaraju R.R., Cogswell M., Das A., Vedantam R., Parikh D., Batra D. Grad-cam: Visual explanations from deep networks via gradient-based localization. Proceedings of the IEEE International Conference on Computer Vision.

